# Multidisciplinary diagnosis and treatment of iatrogenic Cushing syndrome in a patient with obsessive-compulsive disorder

**DOI:** 10.1530/EDM-25-0055

**Published:** 2026-01-29

**Authors:** Ruixue Sun, Shuang Liu, Hong Han, Yunying Cui, Haotian Chen, Kexin Xu, Nan Wu, Yanping Duan, Jing Wei, Xia Hong

**Affiliations:** ^1^ Peking Union Medical College Hospital – Department of Psychological Medicine Beijing, Beijing, China; ^2^ Peking Union Medical College Hospital – Emergency Department Beijing, Beijing, China; ^3^ Peking Union Medical College Hospital – Endocrinology Department Beijing, Beijing, China; ^4^ Peking Union Medical College Hospital – Dentistry Department Beijing, Beijing, China; ^5^ Peking Union Medical College Hospital – Department of Orthopedic Surgery Beijing, Beijing, China; ^6^ Peking Union Medical College Hospital, Beijing, China

**Keywords:** iatrogenic Cushing syndrome, obsessive-compulsive disorder, multidisciplinary diagnosis and treatment, consultation liaison psychiatry, general hospital

## Abstract

**Summary:**

Iatrogenic Cushing syndrome caused by topical corticosteroid preparations is only reported in infants. We describe a case in a patient with obsessive-compulsive disorder: the patient had tic disorder and obsessive-compulsive disorder, and the condition was under stable control. After the appearance of oral ulcers, the patient uncontrollably used a large amount of chlorhexidine dexamethasone membranes, resulting in iatrogenic Cushing syndrome. The fatty liver and abnormal liver function associated with Cushing syndrome led to the discontinuation of fluvoxamine and aripiprazole for obsessive-compulsive disorder. The patient’s tic and compulsive symptoms worsened, and he repeatedly bit his tongue. Under multidisciplinary diagnosis and treatment in emergency department, endocrinology department, psychological department, and dentistry department, both the mental and physical symptoms were controlled and the patient’s prognosis was satisfactory. Genetic testing revealed no clear abnormalities that could explain the patient’s phenotype. Therefore, medication use on obsessive-compulsive disorder patients with somatic diseases should be monitored. Multidisciplinary cooperation, especially consultation liaison psychiatry in general hospitals, is essential for the diagnosis and treatment of patients with both physical and mental symptoms in general hospitals.

**Learning points:**

- It is necessary to monitor medication use when treating OCD patients with somatic diseases.
- Multidisciplinary diagnosis and treatment in general hospitals are advantageous or even the only option for improving the prognosis of comorbid mental disorders and somatic diseases.
- Consultation liaison with psychiatry should be strengthened in general hospitals, especially in the diagnosis and treatment of diseases that are often complicated with psychological symptoms, such as in the endocrinology department.

## Background

Cushing syndrome is an endocrinological disorder that results from abnormally high plasma cortisol levels. Endogenous release of cortisol from adrenal gland tumors or pituitary adenomas can lead to high plasma cortisol levels and Cushing syndrome, and exogenous cortisol from corticosteroid medication use can also lead to iatrogenic Cushing syndrome (ICS) (1). ICS commonly arises from the excessive use of oral and parenteral corticosteroids, and several cases of infants developing ICS after the use of topical preparations have been reported (2). However, ICS secondary to topical corticosteroids in adolescents or adults has not been reported previously according to a PubMed literature search on May 1, 2024. Here, we describe a case of ICS caused by topical steroids in a patient with obsessive-compulsive disorder (OCD). Due to the occurrence of Cushing syndrome complications, the patient’s condition is critical and there is a contradiction between the treatment of mental and physical illnesses. Through multidisciplinary collaboration, patients have been able to recover.

## Case presentation

### Presentation

A 17-year-old male presented with a 3-month history of weight gain and purple striae for 3 months and a 2-week history of headache and nausea.

### Past history

At the age of 6 years, the patient was diagnosed with tic disorder because of throat clearing, blinking, and mouth licking. The symptoms gradually progressed to uncontrollable repetitive limb activity. Due to comorbid OCD, he was treated with fluvoxamine 150 mg/d and aripiprazole 10 mg/d in a psychiatric facility. His condition was well controlled under regular follow-up, and he could study and socialize normally.

### Present history

In September 2023, the patient developed an oral ulcer. He could not control repeated licking of the ulcer surface, and he applied a chlorhexidine dexamethasone membrane (ingredients included chlorhexidine hydrochloride, vitamin B2, dexamethasone sodium phosphate, and dyclonine hydrochloride) to the affected area up to 60–80 times a day. Because of obsessive worries of infection, he frequently changed the membrane to fit it to the wound perfectly. He did not swallow the membrane. In October 2023, the patient gradually developed a round, red face, weight gain, and purple striae. He licked the wound more frequently, and sometimes, he bit his tongue uncontrollably.

On February 9, the patient went to the emergency department because of headache and nausea. At this time, he had reduced the use of chlorhexidine dexamethasone membranes to only occasionally.

### Physical examination

The patient exhibited stable vital signs and clear consciousness. The examination revealed a profound Cushing appearance, with a height of 178 cm, a weight of 115 kg (an increase of approximately 40 kg over the past 4 months), a body mass index of 36.3 kg/m^2^, a full moon face, a buffalo hump, and a thin skin with wide purple striae. Other positive signs included a 2 cm longitudinal wound on the tip of the left tongue and involuntary movement of the mouth and tongue.

## Investigation

Routine blood and blood gas analysis results were generally normal. Lactic acid (LA) was high, at 7.3 mmol/L. Electrolyte levels were as follows – Na: 137 mmol/L; K: 3.2 mmol/L. Liver function parameters were as follows – alanine aminotransferase (ALT): 230 µ/L; aspartate aminotransferase (AST): 151 µ/L; gamma-glutamyl transferase (GGT): 1,116 U/L; lactate dehydrogenase (LDH): 1,004 U/L; total bilirubin (TBil): 29.4 μmol/L; direct bilirubin (DBil): 13.6 μmol/L; and indirect bilirubin (IBil): 15.8 μmol/L. Blood lipid levels were as follows – total cholesterol (TC): 7.46 mmol/L; triglycerides (TGs): 10.52 mmol/L; high-density lipoprotein cholesterol (HDL-C): 0.25 mmol/L; low-density lipoprotein cholesterol (LDL-C): 4.20 mmol/L; and non-HDL-C: 7.21 mmol/L. Hormone levels were as follows – F: < 0.50 μg/dL; adrenocorticotropic hormone (ACTH): 4.4 pg/mL; insulin-like growth factor 1 (IGF-1): 98 ng/mL; and growth hormone (GH): < 0.05 ng/mL. Sex hormones were as follows – testosterone (T): 1.20 ng/mL; follicle-stimulating hormone (FSH): 2.23 IU/L; and luteinizing hormone (LH): 1.19 IU/L.

Thyroid function was as follows – third-generation thyroid-stimulating hormone (TSH3): 7.314 μIU/mL; free thyroxine (FT4): 1.75 ng/dL; and free triiodothyronine (FT3): 4.00 pg/mL. Head magnetic resonance imaging (MRI) + susceptibility weighted imaging (SWI) findings were normal.

Thoracic and abdominal computed tomography (CT) showed severe fatty liver and bilateral thin adrenal glands.

## Treatment

The patient was diagnosed with hyperlactatemia caused by abnormal liver function secondary to severe hepatic adipose infiltration in the emergency department. Hyperlipidemia was treated with intravenous pumping of insulin, and hepatoprotective drugs were used to improve liver function. To avoid further deterioration of liver function, the use of fluvoxamine and aripiprazole was stopped.

Endocrinologists consulted the patient and diagnosed him as having ICS. The endocrinologists discontinued external application of the compound chlorhexidine dexamethasone membrane and administered 100 mg/d hydrocortisone for replacement therapy to prevent adrenal insufficiency.

After the discontinuation of fluvoxamine and aripiprazole, the patient bit his tongue more frequently, causing partial tongue loss. He became fidgety and felt unbearable pain. Physicians in the psychological medicine department considered that the patient was experiencing an exacerbation of obsessive-compulsive symptoms accompanied by anxiety. As liver function improved, the use of fluvoxamine was resumed, with dosages gradually increased from 25 to 100 mg qd, and aripiprazole use was resumed, with dosages increased from 2.5 to 5 mg qd.

Stomatologists prescribed external use of bovine basic fibroblast growth factor to promote wound healing and dyclonine mucilage to relieve pain.

During this period, the patient’s condition was complicated by influenza A infection, which was treated with oseltamivir.

## Outcome and follow-up

After multidisciplinary treatment, the patient’s headache and nausea resolved. His mood was stable, and he stopped biting his tongue but still shouted uncontrollably. His liver function improved, and his lactate and blood lipid levels decreased – ALT: 93 U/L; AST: 103 U/L; GGT: 577 U/L; LDH: 351 U/L; TBil: 23.3 μmol/L; DBil: 17.6 μmol/L; and LA: 3.0 mmol/L. His blood lipid levels were as follows– TC: 5.81 mmol/L; TGs: 6.34 mmol/L; HDL-C: 0.66 mmol/L; and non-HDL-C: 5.15 mmol/L. F and ACTH levels rebounded to 4.0 μg/dL and 22.4 pg/mL, respectively.

As the patient’s condition improved, doctors in the emergency department transitioned the medication regimen to oral liver protection and lipid-lowering agents and gradually reduced the dose of hydrocortisone. Then, the patient was discharged after approximately 1 month of hospitalization.

At the 1-month follow-up, the doses of fluvoxamine and aripiprazole were gradually increased to 150 mg/d and 10 mg/d, respectively, while liver function was closely monitored. The patient’s obsessive-compulsive symptoms were relieved, and the patient lost 15 kg of weight by controlling his diet and exercising, after which he was ready to return to school.

At the end, we had several questions: was the patient sensitive to glucocorticoids? Was the fatty liver so severe that it led to hyperlactatemia? Given his childhood tic symptoms, we considered the possibility of genetic abnormalities. We performed whole exome capture and sequencing of the genomic DNA of the patient and analyzed single nucleotide variations, small fragment insertion and deletion variations, and large fragment copy number variations based on second-generation sequencing data. Although the results showed variations in four genes, which include *GYS2*, *LMNA*, *SCN2A*, and *SETD1A*, they are difficult to explain the patient’s phenotype and do not have clinical significance (3, 4). Perhaps, the patient’s condition was purely caused by excessive intake of glucocorticoids.

## Discussion

OCD is one of the most common psychiatric illnesses and the most impairing non-communicable disease in adults worldwide (5). OCD has a profound negative impact on the quality of life and functioning of affected individuals and their families and imposes substantial costs at the societal level (6). In this case, the patient’s obsessive-compulsive symptoms even led to excessive use of topical corticosteroids and the complications of the resulting ICS were almost life-threatening. Therefore, we focused on the association between OCD and substance misuse.

The ventral affective circuit, which is one of the neurobiological models of OCD, is thought to be involved in reward processing (7). Reward processing may be associated with substance use disorders (SUDs). However, whether OCD is a risk factor for a greater incidence of SUDs remains unclear. In 2022, Virtanen’s cohort study revealed that obsessive-compulsive symptoms at 18 years of age were associated with increased symptoms of alcohol dependence and drug dependence (8). This finding at least challenged the notion that OCD is protective against developing substance misuse. Combined with our case, close monitoring for substance or medicine use disorder is necessary in the management of patients with both OCD and somatic diseases.

In this case, the misused substance was a corticosteroid, which is related to ICS. Currently, definitive criteria or highly sensitive and specific diagnostic tests for ICS are lacking. Neonates and infants are particularly at risk of ICS caused by topical corticosteroids because of their physiology (9). Similarly, the skin barrier of our patient might have been disrupted because of the ulcer. Cushing syndrome is associated with psychiatric and neurocognitive disorders through the induction of brain damage. Major depression, mania, anxiety, and neurocognitive impairment are the most important clinical abnormalities observed in patients with Cushing syndrome (10). This association of Cushing syndrome with psychiatric disorders, as well as somatic disease maladjustment, could explain the patient’s anxiety. Although there are no reports about obsessive-compulsive symptoms being directly induced by Cushing syndrome, anxiety can aggravate preexisting obsessive-compulsive symptoms. Therefore, a patient’s physical condition and mental symptoms should both be accounted for during treatment.

Several difficulties were encountered in the diagnostic and treatment processes. First, in the initial laboratory test, the patient presented with hyperlactatemia, which is commonly a life-threatening indication. Physicians in the emergency department rapidly determined that the etiology of hyperlactatemia was severe hepatic adipose infiltration and stabilized the patient’s circulation state, which shortened the length of stay in the resuscitation room. In the general ward, family assistance was allowed and was beneficial for the patient’s emotional stability. Second, ICS secondary to topical corticosteroids in adolescents or adults is rare, and the endocrinologists made a clear diagnosis based on the patient’s characteristic signs of Cushing syndrome, medication history, hormone levels, and negative imaging results and stopped the continued intake of exogenous corticosteroids. Third, how to resume OCD treatment in patients with impaired liver function is challenging. Fortunately, an emergency doctor treated hyperlipidemia with insulin and liver function improved in three days, providing conditions conducive to the resumption of fluvoxamine and aripiprazole. Finally, reducing tongue injury and pain was an urgent matter when the symptoms of tics and obsessions were uncontrolled. The therapy prescribed by physicians in the stomatology department helped heal the wound and partly relieved the patient’s pain. Therefore, the cooperation of multiple departments was the key to improving the condition of this patient. Consultation liaison psychiatry service has started only recently in China, with most psychiatric departments in general hospitals having just been established in recent years. This case also reflects the importance of developing the consultation liaison psychiatry service in our country.

## Declaration of interest

There is no conflict of interest that could be perceived as prejudicing the impartiality of the research reported.

## Funding

This research did not receive any specific grant from any funding agency in the public, commercial, or not-for-profit sector.

## Patient consent

Written informed consent for publication of their clinical details was obtained from the patient.

## Author contribution statement

RS organized the case information and wrote the manuscript. SL, HH, YC, HC, YD, and JW participated in the diagnosis and treatment and verified the relevant professional information in the articles. KC and NW conducted genetic testing result analysis. XH proposed the article’s ideas and made overall revisions to the manuscript.

## Patient’s perspective

At the beginning, I wanted to make a film for the treatment of oral ulcer smooth. As the film was uneven, I felt very awkward and had to stick it many times. Later, I was always hungry, ate a lot of things, and gained dozens of kilograms. I went to the hospital to get checked; my liver function was very poor, and I could not take medicine to treat my compulsion. I was very upset and could not help biting my tongue. My tongue was broken and painful, but I still could not help biting. After my liver function was treated, I could take the original medicine, my mood calmed down, I slowly controlled my diet, I played ball and exercised, my weight basically returned to normal, and I went back to school. In the future, when I use any medicine, I will remind myself and my doctor that I am still an obsessive-compulsive patient. I do not want to have this painful experience again.
